# Editorial: Proteomics in atherosclerosis

**DOI:** 10.3389/fcvm.2026.1792281

**Published:** 2026-03-27

**Authors:** Xi-Ming Yuan, Sasha A. Singh

**Affiliations:** 1 Occupational and Environmental Medicine, Department of Health, Medicine and Caring Sciences, Linköping University, Linköping, Sweden; 2 Center for Interdisciplinary Cardiovascular Sciences, Division of Cardiovascular Medicine, Department of Medicine, Brigham Women’s Hospital, Harvard Medical School, Boston, MA, United States

**Keywords:** atherosclerotic cardiovascular disease, biomarker, coronary heart disease, proteomics, transcriptomics

## Atherosclerotic cardiovascular diseases and risk biomarkers

Atherosclerotic cardiovascular disease (ASCVD) remains the leading cause of mortality worldwide and continues to impose a substantial burden of morbidity despite therapeutic advances. Ongoing research into its underlying mechanisms and new risk biomarkers is essential to further mitigate this global health challenge. Without new preventive and therapeutic strategies, population aging and the global risk factors such as obesity, diabetes, and sedentary lifestyle are expected to keep ASCVD rates high (1).

Traditional risk scores based on age, blood pressure, lipids, smoking, and diabetes do not fully capture individual risk, especially in younger people, women, and different ethnic groups. New approaches using genetics, multi-omics, and machine learning aim to move beyond simple “high/low risk” categories to more precise, prediction models, which requires further biomarker research (2).

Even with LDL-cholesterol lowering and guideline-directed therapies, patients often retain a considerable “residual risk” of heart attack, stroke, and cardiovascular death (3). This residual risk is linked to factors such as triglyceride-rich lipoproteins, inflammation, thrombosis, and other pathways not fully addressed by current drugs, which motivates ongoing research for novel biomarkers (4, 5).

Long-running prospective community-based initiatives, such as the Framingham Heart Study (FHS, 1948-present) (6) and the Atherosclerosis Risk in Communities Study (ARIC, 1985-present) (7), have accordingly updated aims to integrate genetic profiling and multi-omics to identify novel cardiovascular disease risk factors and biomarkers. More recent prospective studies have cohorts of a half-million (UK Biobank, 2003-present) to million (Million Veteran Program, USA, 2011-present) in order to investigate the effects of genetic, lifestyle and environmental factors at large population scale (8, 9).

Proteomics has revolutionized the search for biomarkers in atherosclerosis, enabling the identification of protein signatures that reflect disease presence, progression, and risk. By analyzing tissues, blood, and other biological samples, proteomic approaches provide a wide-ranging molecular view in risk stratification, early diagnosis, and the development of targeted therapies (10, 11).

## Contributor highlights

Our call for submissions to Research Topic *Proteomics in Atherosclerosis* is timely given the recent announcement of the UK Biobank Pharma Proteomics Project Consortium, aiming to establish connections between genetic variants and their corresponding protein products (12).

In our topic Research Topic, the paper by Chen et al. investigates whether *plasma proteomic profiles* can predict carotid intima-media thickness (cIMT), using data from 6,136 UK Biobank participants. The study is methodologically robust, offering predictive value of proteomics over traditional risk factors. While the improvement in prediction is modest, the findings pave the way for *early vascular disease detection*.

Zhang et al. investigates clinical characteristics and proteomic biomarkers in *young patients* (<45 years) with *acute coronary syndrome* (ACS) compared to middle-aged and elderly patients. The study focuses on premature ACS, a growing concern due to lifestyle changes. It integrates clinical risk factors with proteomic analysis using the proximity extension assay on the Olink platform, highlighting age-specific biomarker patterns among young patients with ACS. While preliminary, these findings underscore the need for age-specific prevention strategies and biomarker-driven diagnostics.

The paper by Hou et al. aims to elucidate the *plasma proteomic profile* of patients with *in-stent restenosis* (ISR) after drug-eluting stent implantation using Tandem Mass Tag (TMT)-based quantitative proteomics. The global plasma proteome analysis of ISR patients identifies candidate biomarkers and pathways potentially involved in ISR pathogenesis. The study combines high-throughput proteomics with bioinformatics and ELISA validation. The study is the first comprehensive plasma proteome map for ISR. While primary, the study identifies potential biomarkers and pathways that could lead to early diagnosis and novel therapeutic strategies for ISR.

Atrial fibrillation (AF) and atherosclerosis are closely connected, sharing risk factors such as aging, hypertension, and diabetes. Atherosclerosis independently increases the risk of AF, while AF raises the likelihood of stroke and arterial thrombosis. Tu et al. investigates *acetylated proteomics* in left atrial appendage tissues from patients with *valvular heart disease*, comparing those with chronic *atrial fibrillation* (AF) to those in sinus rhythm. The authors did full mapping of acetylated proteins in human atrial tissue during AF. The study links lysine acetylation to metabolic and contractile remodeling in AF, suggesting new therapeutic targets, improving energy supply and contractility. The study also indicates that acetylation modulates key enzymes in energy metabolism including fatty acid *β*-oxidation enzymes, contributing to AF progression. The identified acetylation sites may serve as diagnostic or prognostic markers for AF.

The paper by Elkenani et al. investigates molecular mechanisms underlying *paradoxical aortic stenosis* using cellular and extracellular proteomic profiling of left ventricular biopsies. It highlights distinct molecular signatures, including Ca^2+^ signaling dysregulation, oxidative stress markers, and extracellular Cyclophilin A (CyPA). This study is methodologically innovative and clinically relevant, offering new insights into pathophysiology of aortic stenosis. The identification of CyPA as a potential extracellular mediator and the dysregulation of Ca^2+^ handling proteins highlight potential targets for future therapeutic strategies.

The study by Okui et al. investigates the metabolic consequences of Carnitine O-octanoyltransferase (CROT) deficiency in normolipidemic mice, focusing on liver and plasma metabolomes. The authors suggest that CROT activity influences fatty acid partitioning and possibly vascular calcification via lipid signaling. Omega-3 fatty acids and azelaic acid may serve as potential biomarkers of CROT inhibition.

The study by Brandes et al. demonstrates that specific microRNAs are simultaneously expressed in *circulating extracellular vesicles* (EVs) and *atherosclerotic plaques* in patients with carotid artery stenosis. This paired analysis is novel and suggests that circulating EVs reflect plaque development in patients with symptomatic carotid artery stenosis, which can serve as biomarker candidates for detecting the presence of atherosclerotic plaques.

Circulating apolipoproteins (APOA1, APOE) whose lipid binding and metabolizing functions are consequential to ASCVD risk, were recently discovered to be ADP-ribosylated. Kasai et al. provide a behind the scenes view of mass spectrometer acquisition optimizations required to characterize the posttranslational modification (PTM), *ADP-ribosylation*. The consequences of this PTM on apolipoprotein function and ASCVD risk may entail mechanistic and functional studies on the chemistry of ADP-ribosylated peptides (13).

Finally, while proteins are often in the spotlight as potential biomarker or therapeutic targets, the review by Yan et al. provides an overview of miRNAs research in the context of coronary heart disease and diabetes in preclinical and clinical settings.

Overall, the studies in this collection offer important insights into risk stratification, biomarker-guided diagnostics, and therapeutic strategies for ASCVD. Although differences in study design prevent us from identifying common core molecular mechanisms across all articles, several proteins emerge as potential drivers of atherosclerosis progression. These include cytoplasmic protein NCK1 in relation to carotid intima–media thickness; growth differentiation factor 15, osteopontin, and NT-proBNP in patients with acute coronary syndrome; and fetuin-B, apolipoprotein C-III, and cholesteryl ester transfer protein in relation to in-stent restenosis.
